# Physically informed machine-learning algorithms for the identification of two-dimensional atomic crystals

**DOI:** 10.1038/s41598-023-33298-6

**Published:** 2023-04-15

**Authors:** Laura Zichi, Tianci Liu, Elizabeth Drueke, Liuyan Zhao, Gongjun Xu

**Affiliations:** 1 Department of Physics, University of Michigan, Ann Arbor, 48109 USA; 2 Department of Statistics, University of Michigan, Ann Arbor, 48109 USA

**Keywords:** Condensed-matter physics, Graphene

## Abstract

After graphene was first exfoliated in 2004, research worldwide has focused on discovering and exploiting its distinctive electronic, mechanical, and structural properties. Application of the efficacious methodology used to fabricate graphene, mechanical exfoliation followed by optical microscopy inspection, to other analogous bulk materials has resulted in many more two-dimensional (2D) atomic crystals. Despite their fascinating physical properties, manual identification of 2D atomic crystals has the clear drawback of low-throughput and hence is impractical for any scale-up applications of 2D samples. To combat this, recent integration of high-performance machine-learning techniques, usually deep learning algorithms because of their impressive object recognition abilities, with optical microscopy have been used to accelerate and automate this traditional flake identification process. However, deep learning methods require immense datasets and rely on uninterpretable and complicated algorithms for predictions. Conversely, tree-based machine-learning algorithms represent highly transparent and accessible models. We investigate these tree-based algorithms, with features that mimic color contrast, for automating the manual inspection process of exfoliated 2D materials (e.g., MoSe_2_). We examine their performance in comparison to ResNet, a famous Convolutional Neural Network (CNN), in terms of accuracy and the physical nature of their decision-making process. We find that the decision trees, gradient boosted decision trees, and random forests utilize physical aspects of the images to successfully identify 2D atomic crystals without suffering from extreme overfitting and high training dataset demands. We also employ a post-hoc study that identifies the sub-regions CNNs rely on for classification and find that they regularly utilize physically insignificant image attributes when correctly identifying thin materials.

## Introduction

Since the first realization in 2004 that graphite could be mechanically exfoliated into graphene in ambient conditions using a simple piece of Scotch tape^1^, the study of graphene in the two-dimensional (2D) limit has demonstrated a rich landscape for interesting physical phenomena, ranging from Dirac electrons in graphene^2^ to unconventional superconductivity in twisted graphene moire superlattices^3^. This simple yet effective methodology used for fabricating graphene, scotch tape peeling followed by optical microscope imaging, has been exploited to greatly expanded the 2D atomic crystal pool, discovering 2D transition metal dichalcogenide (TMD) semiconductors^4^ 2D magnets^5^ and many on. However, many of these materials show deterioration in ambient conditions^6–8^. This has prompted researchers to innovate fabrication environments that prevent degradation of samples. Further improvements to current fabrication and visualization techniques to produce large 2D materials remain imperative for their fundamental research and commercial-level applications in next generation electronics, optoelectronics, and energy storage^9,10^.

Isolation of 2D materials involves cleaving the bulk material on a wafer, usually oxidized Si. Current visualization techniques, including atomic-force, scanning tunneling, and electron microscopies, exhibit low-throughput of locating the resultant relevant thin materials of only several nm in diameter known as flakes^11^. Raman Microscopy, which can realize accurate 2D structures, has not been automated and relies on experienced users^12^. With the recent surge of successful high-performance machine-learning algorithms for object classification within images, many have applied these methods to locate exfoliated 2D materials in optical images^13–16^. Usually the machine-learning algorithms used for flake identification are deep neural networks due to their great success with object recognition^17–20^. The integration of machine-learning and optical microscopy techniques can accelerate flake identification. This can then expedite innovations within the flake fabrication process to promote practical 2D material applications and research.

Although the neural networks attain high accuracies, their high computational complexity and large dataset requirements can render them difficult to employ in laboratory settings which require manual collection of training data. Furthermore, critiqued as “black boxes”, no comprehensive theoretical understanding of neural networks’ inner layers exists^21^. In certain environments, accuracy can be sacrificed for more accessible and transparent algorithms. Therefore, we propose coupling optical microscopy techniques with tree-based algorithms as an alternative to deep learning methods for a more accessible and transparent method for accelerating the identification of 2D materials. We employed, for comparison, tree-based methods—decision trees, gradient boosted decision trees and random forests—and deep Convolutional Neural Networks (CNNs) for identification of exfoliated MoSe_2_ under different optical settings. The tree-based algorithm’s features mimicked the physical method of identifying flakes using color contrast, a technique currently used throughout the 2D materials community, giving them a more understandable physical motivation than the CNNs^11^. We compare the physicality of these algorithms through tree visualizations and Gradient-weighted Class Activation Mapping (Grad-CAM), a post-hoc study that identifies the sub-regions CNNs rely on for classification, and their accuracies to understand their potential application^22,23^. We find that the CNN’s fortuitous ability to locate 2D atomic crystals when correctly classifying images emphasizes their unphysical and opaque decision-making process.

## Methods

**Figure 1:**
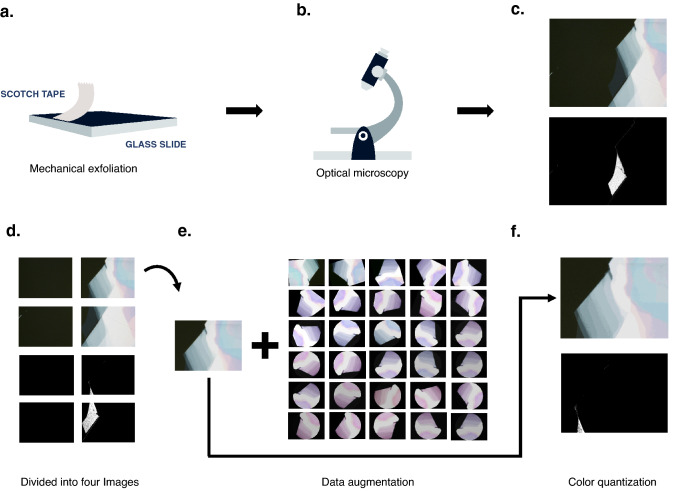
MoSe_2_ flake fabrication and image collection and processing. (**a**) Mechanical exfoliation of MoSe_2_ with scotch tape to produce flakes which are then (**b**) imaged with optical microscopy. (**c**) A typical optical image of a flake and surrounding bulk material with a masked version of the image below which only displays the flake in white. (**d**) The four resulting images when the original image in (**c**) is divided with the masked version below. (**e**) The resulting 30 images produced through the augmentation methods of padding, rotating, flipping, and color jitter. (**f**) The image recreated with 20 colors again with the masked version below.

Optical images used in this study include transition metal dichalcogenide (TMD) flakes on SiO_2_/Si substrate, TMD flakes on Polydimethylsiloxane (PDMS), and TMD flakes on SiO_2_/Si and PDMS (if any). The usage of multiple types of substrates models more realistic flake fabrication environments and strengthens algorithm robustness. All these samples were mechanically exfoliated in a 99.999% N_2_-filled glove box (Fig. 1a). The optical images were also acquired in the same environment with no exposure to ambient conditions occurring between fabrication and imaging processes (Fig. 1b). The 83 MoSe_2_ images used throughout this study were taken at the 100× magnification by various members of the Hui Deng group who selected different amounts of light to illuminate the sample (Fig. 1c). These images are divided into four smaller symmetric images containing randomized amounts of flake and bulk material which were then manually reclassified (Fig. 1d).

The extremely time-consuming process of locating a flake renders these datasets small, a common occurrence in many domains such as medical sciences and physics. However, deep learning models, such as CNNs, usually contain numerous parameters to learn and require large-scale data to train on to avoid severe overfitting. Data augmentation is a practical solution to this problem^24^. By generating new samples based on existing data, data augmentation produces training data with boosted diversity and sample sizes, on which better performing deep learning models can be trained (see Supplementary methods). The benefit of applying data augmentation is two-fold. First, it enlarges the data that CNNs are trained on. Second, the randomness induced by the augmentation of the data encourages the CNNs to capture and extract spatially invariant features to make predictions, improving the robustness of the models^24^. In fact, augmentation is quite common when using CNNs even with large datasets for this reason. Typically, different augmented images are generated on the fly during the model training period, which further helps models to extract robust features. Due to limited computing resources, we generated augmented data prior to fitting any models, expanding the data from 332 to 10,292 images (Fig. 1e).

Once augmented, we applied color quantization to all images (Fig. 1f). The quantization decreased noise and image colors to a manageable number necessary for extracting the tree-based algorithms’ features. The color quantization algorithm uses a pixel-wise Vector Quantization to reduce colors within the image to a desired quantity while preserving the original quality^16^. We employed a K-means clustering to locate the desired number of color cluster centers using a single byte and pixel representation in 3D space. The K-means clustering trains on a small sample of the image and then predicts the color indices for the rest of the image, recreating it with the specified number of colors (see Supplementary methods). We recreated the original MoSe_2_ images with 5, 20, and 256 colors to examine which resolution produced the most effective and generalizable models. Images were not recreated with less than five colors because the resulting images would consist of only background colors and not show the small flake in the original image. Images recreated with 20 colors appeared almost indistinguishable from the original while still greatly decreasing noise. To mimic an unquantized image, we recreated images with 256 color clusters. We compare the accuracies of the tree-based algorithms and CNNs on datasets of our images recreated with 5 and 20 colors. We also compare the tree-based algorithms' performance on our images recreated with 256 colors to the CNNs on the unquantized images (it is not necessary to perform quantization for CNN classification).

After processing the optical images, we employ tree-based and deep learning algorithms for their classification. Tree-based algorithms are a family of supervised machine learning that perform classification or regression based on the value of the features of the tree-like structure it constructs. A tree consists of an initial root node, decision nodes that indicate if the input image contains a 2D flake or not, and childless leaf nodes (or terminal nodes) where a target variable class or value is assigned^25^. Decision trees’ various advantages include the ability to successfully model complex interactions with discreet and continuous attributes, high generalizability, robustness to predictor variable outliers, and an easily interpreted decision-making process^26,27^. These attributes motivate the coupling of tree-based algorithms and optical microscopy for the accelerated identification of 2D materials. Specifically, we employ decision trees along with ensemble classifiers, such as random forests and gradient boosted decision trees, for improved prediction accuracies and smoother classification boundaries^28–30^.

The features of the single and ensemble trees mimic the physical method of using color contrast for identifying graphene crystallites against a thick background. The flakes are sufficiently thin so that their interference color will differ from an empty wafer, creating a visible optical contrast for identification^11^. We calculate an analogous color contrast for each input image. The tree-based methods then use this color contrast data to make their decisions and classify images.

This color contrast for the tree-based methods is calculated from the 2D matrix representation of the input images as follows. The 2D matrix representation of the input image is fed to the quantization algorithm which recreates the image with the specified number of colors. We then calculate the color difference, based on RGB color codes, between every combination of color clusters to model optical contrast. These differences are sorted into different color contrast ranges which encompass data extrema. To prevent model overfitting, especially for the ensemble classifiers, only three relevant color contrast ranges were chosen for training and testing the models: the lowest range, a middle range representative of the color contrast between a flake and background material, and the highest range (see Supplementary methods). This list of the number of color differences in each range is what the tree-based methods use for classification.

Once these features are calculated, we employed a k-fold cross-validation grid search to determine the best values for each estimator’s hyperparameters. The k-fold cross-validation–an iterative process that divides the train data into k partitions–uses one partition for validation (testing) and the remaining k − 1 for training during each iteration^31^. For each tree-based method, the estimator with the combination of hyperparameters which produces the highest accuracy on the test data was selected (see Supplementary methods). We employed a five-fold cross-validation with a standard 75/25 train/test split. After finetuning the decision tree’s hyperparameters with k-fold cross-validation, we produced visualizations of the estimator to evaluate the physical nature of its decisions. The gradient boosted decision tree and random forest estimators represent ensembles of decision trees so the overall nature of their decisions can be extrapolated from a visualization of a single decision tree since they all use the same inherently physical features.

Along with the tree-based methods, we also examined deep learning algorithms. Recently, deep neural networks, which learn more flexible latent representations with successive layers of abstraction, have shown great success on a variety of tasks including object recognition^32,33^. Deep convolutional neural networks take an image as input and output a class label or other types of results depending on the goal of the task. During the feed forward step, a sequence of convolution and pooling operations are applied to the image to extract visuals. The CNN model we employ is a ResNet18^34^, and we train new networks from scratch by initializing parameters with uniform random variables^35^ due to the lack of public neural networks pre-trained on similar data. The training of ResNet18 is as follows. We used 75% original images and all their augmented images as the training. This can further be split into training and validation sets when tuning hyper-parameters. We used a small batch size of 4 and run 50 epochs using stochastic gradient descent method with momentum^36^. We used a learning rate of 0.01 and momentum factor of 0.9. Various efforts work to produce accurate visualizations of the inner layers of CNNs including Grad-CAM which we employed. Grad-CAM does not give a complete visualization of the CNNs as it only uses information from the last convolutional layer of the CNN. However, this last convolutional layer is expected to have the best trade-off between high-level semantics and spatial information rendering Grad-CAMs successful in visualizing what CNNs use for decisions^22^.

## Results and discussions

**Figure 2:**
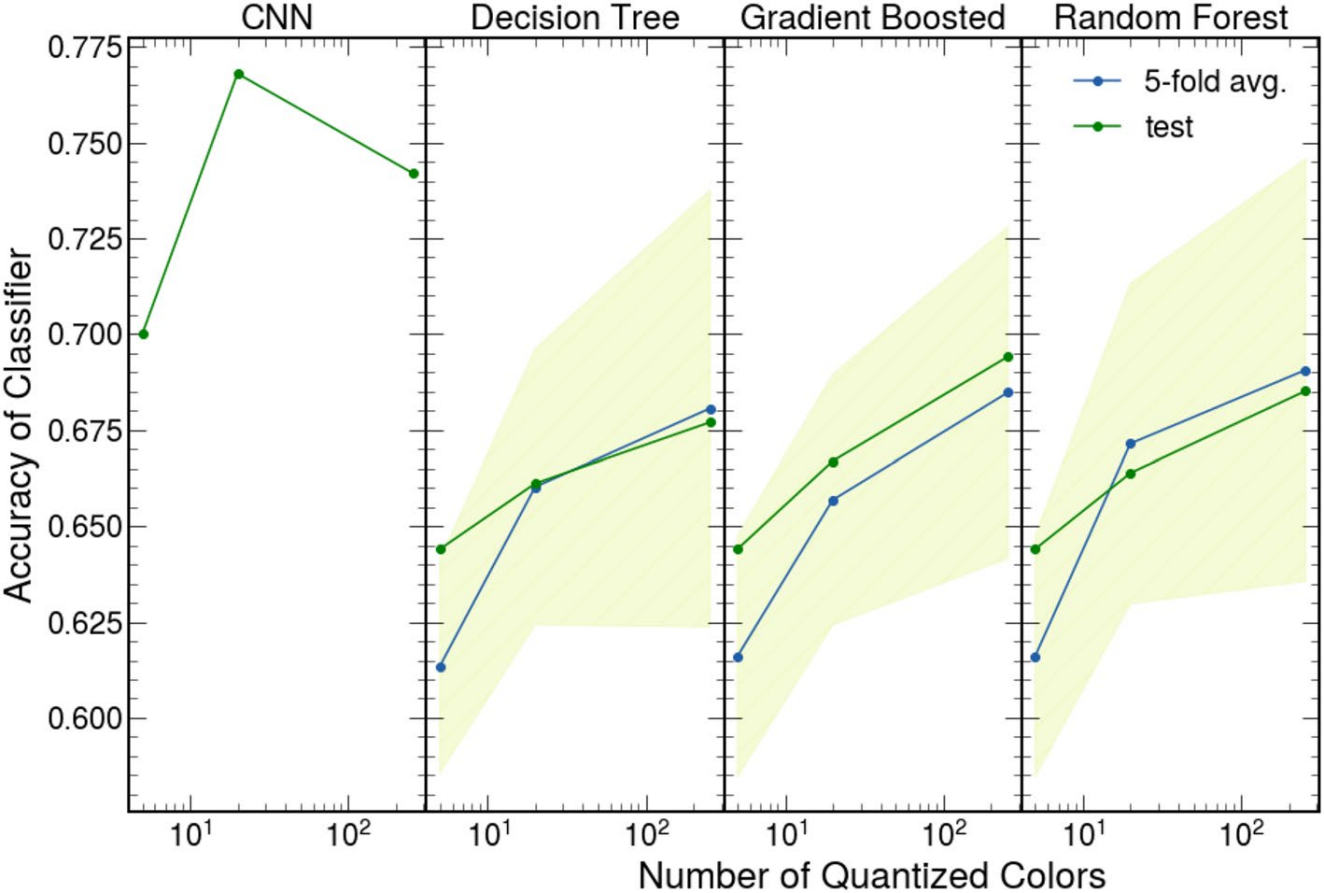
CNN and tree-based machine learning algorithms’ accuracies. The accuracy of CNN and tree-based algorithms depends on the number of color clusters in the recreated image. The tree-based methods and CNNs were trained and tested on images recreated with 5, 20, and 256 colors and 5, 20, and infinite (original image) colors. The CNNs’ accuracies (first panel) determined with a 75/25 train test split. Tree-based algorithms’ accuracies (last three panels) shown as the average test accuracy from the five-fold cross-validation used to select the algorithm's hyperparameters (blue) and test accuracy from 75/25 train test split after this optimization (green). The standard error for the five-fold cross-validation test accuracy is represented by the shaded region.

Both machine-learning methods proved effective for accelerating the identification of thin materials. The accuracies of the tree-based methods are displayed as both the average test score from the five-fold cross-validation (blue) as well as the accuracy on the test dataset (green) as a function of the number of quantized colors (Fig. 2). The CNNs demonstrated higher accuracies than the tree-based methods for every color quantization. They performed with accuracies between 70.0% and 76.0% and showed no discernable dependency on color quantization. Conversely, the test and average five-fold cross-validation accuracy of the tree-based methods improved as the images were quantized with more colors for all three algorithms. Unsurprisingly, the ensemble estimators, gradient boosted decision trees and random forests, performed with the highest accuracies of the tree-based methods. The accuracy of these methods ranged from 64.5 to 69.5% (Fig. 2).

Although the CNNs achieved higher accuracies, they represent opaque algorithms that lack accessibility because of their high computational and dataset requirements. The CNNs showcased severe overfitting which led to relatively poor performance on unseen datasets. This is showcased by examining the performance of the CNNs when trained with smaller training datasets. When trained with 10% and 50% of all training data from a 75/25 train test split, the CNNs suffered a large loss of accuracy (up to 20%) and showcased signs of extreme overfitting (training accuracies range from 96 to 100%). Conversely, the tree-based methods maintained their performance (up to 6% loss in test accuracy) without severe overfitting (training accuracies between 61 and 87%). This emphasizes their accessibility when working with limited data and diverse images (see Supplementary methods). The tree-based methods showcase further accessibility in terms of computational time. To fit the algorithms, the CNNs require hours while the tree-based methods take a few seconds (see Supplementary methods).

**Figure 3:**
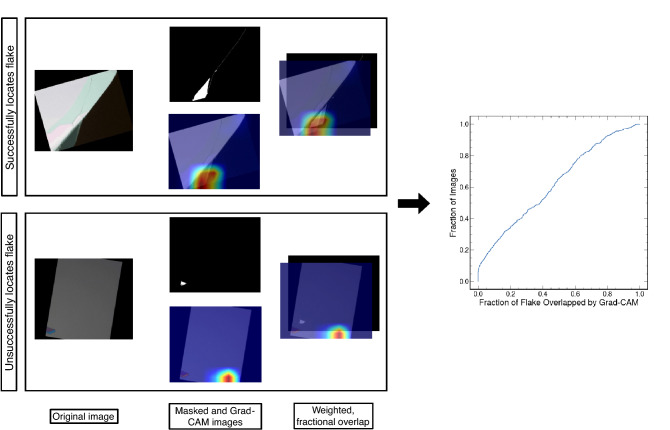
Grad-CAM Evaluation with an Empirical Cumulative Distribution Function. For 500 correctly classified flake images quantized with 20 colors, the weighted fraction of flake highlighted by the Grad-CAM heatmap is displayed as an empirical cumulative distribution function (on the right). The process for determining the fractional overlap for an unsuccessful image (overlap < 0.1) and a successful image (overlap > 0.9) are shown. In both instances, a masking algorithm locates the thin flake and determines the weighted fraction of overlap between this flake and the Grad-CAM heatmap.

Moreover, visualizations of the subregions the CNNs used for classification, through Grad-CAM images, indicate that the decision processes may lack physical integrity. Intuitively, the Grad-CAM uses gradients of the label *flake* to features (pixels) to locate and visualize the image subregions that the CNN used for training and testing during classification. The final convolutional layer is used to construct a coarse heatmap indicating these subregions which is then overlaid onto the original image. We evaluated the Grad-CAM’s ability to locate the flakes in 500 correctly classified images quantized with 20 colors with flakes to ascertain the physical nature of the CNNs. A masking algorithm located the region of each image containing a flake (see Supplementary methods). We then summed the Grad-CAM heatmap’s weights (which are normalized between zero and one) in this region and divided by the total flake area. We summarized the results of this evaluation as an empirical cumulative distribution function (ECDF) shown in Fig. 3. We also showcase in Fig. 3 an example of a successful Grad-CAM image with an overlap fraction of 0.95 along with an unsuccessful Grad-CAM image with 0.00 fractional overlap. The ECDF’s median of 0.4 fractional overlap between a flake and a Grad-CAM image indicates that the CNN’s did not use regions near the flake for training and testing. Instead, the CNN’s regularly failed to locate the flake, training and testing on other potentially meaningless image features while still correctly classifying images (Fig. 3). The CNN's fortuitous ability to locate flakes emphasizes the need for caution when blindly applying these high-performance deep learning algorithms.

**Figure 4:**
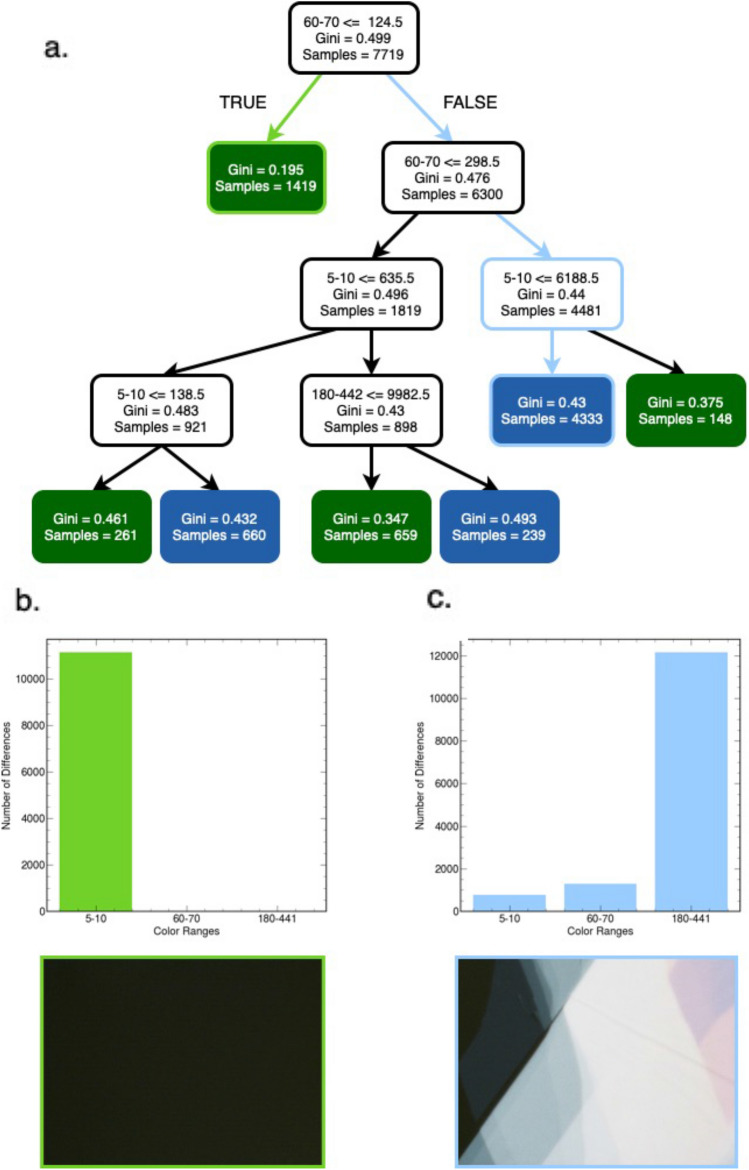
Visualization of decision tree after training. (**a**) Visualization of a decision tree classifier after training with 256 color clusters with a train and test accuracy of 73% and 67%. Each node shows the feature (number of color cluster differences in each range) used to indicate if the input image contains a 2D flake or not, the Gini impurity (a measure of the probability of misclassifying a random element when randomly classifying it based on the class distribution), the node's samples, and classification if a terminal node (blue and green as flake and no flake). (**b**) An example of the tree traversal of an image without a flake and associated bins (colored light green) and (**c**) an image with a flake and associated bins (colored light blue).

Conversely, the tree-based algorithms inherently relied on physical image attributes because their features are based on color contrast. Visualizations of the tree-based methods’ decisions demonstrate their reliance on the physical color contrast of flake to bulk or background material for classification. To better highlight these ideas, we showcase how an image with a flake and without a flake are classified based on a decision tree trained on data quantized with 256 color cluster centers in Fig. 4. Each image’s associated features of number of color differences in various ranges are shown as a bar chart below the images. The image without a flake traverses left from the leaf node and is classified as not containing a flake by a terminal node (Fig. 4b). Starting from the root node, the image with a flake traverses the tree to the right until being classified as containing a flake at a terminal node (Fig. 4c). The tree-based methods used the number of large color differences between pixels to identify images with flakes surrounded by bulk material (Fig. 4b). Similarly, these methods regularly used high numbers of low color differences to correctly classify optical images that are almost all background with little MoSe_2_ as not containing flakes (Fig. 4c). Furthermore, the training accuracies ranged from 61 to 73% indicating little to no overfitting. However, the high Gini impurities associated with the various tree decision nodes indicate a lack of confidence in classification, highlighted by the tree-based estimator’s lower accuracies.

These lower accuracies result from high rates of false negatives as indicated by the confusion matrices (see Supplementary methods). A manual post image processing revealed that the false negatives usually contained very small flakes. Further evaluation of the tree-based methods with operating characteristic (ROC) curves indicated that by tuning the true positive and false positive rates the tree-based methods can increase throughput of manual flake identification by a factor of three (see Supplementary methods). Furthermore, the most successful tree-based classifier, the gradient boosted decision trees trained with 256 colors, achieved a testing accuracy of 70%. Adaption of this classifier during the identification process of the 2D materials would greatly accelerate locating flakes. Although tree-based methods require fine-tuning of features to increase accuracies, they represent a promising physically informed and transparent alternative to deep-learning algorithms for coupling with optical microscopy for rapid identification of thin materials. In future work, the features of the tree-based methods can be further tuned to produce higher accuracies and other methods such as unsupervised learning could be employed for classification of 2D materials.

## Supplementary Information

Supplementary Information.

## Data Availability

Optical images used for machine-learning training and testing are available upon reasonable request. All codes discussed here (machine-learning methods, masking algorithm, and Grad-CAM evaluation) are available on GitHub at https://github.com/lzichi/Thin-Materials-ML.
