# Supplementary material for: Physically informed machine-learning algorithms for the identification of two-dimensional atomic crystals
Source: Sci Rep. 2023 Apr 15;13:6143. doi: 10.1038/s41598-023-33298-6 (PMC10105744; doi:10.1038/s41598-023-33298-6)
Supplement: Supplementary file 1 — Supplementary Information. [file 41598_2023_33298_MOESM1_ESM.docx]

Data Augmentation

In this paper, four types of augmentation methods were applied sequentially to augment image data: padding, rotating, flipping, and color jitter. Augmented images have the same labels as the original images. The rationale of these augmentation methods is guaranteed by the belief that information from each image has some types of translation invariance. For instance, rotating or flipping an image does not change the existence of a flake or not. However, a dramatic modification of the brightness, contrast, or hue of an image (jitter) can remove flakes from images. To avoid mislabeling induced by data augmentation, we restricted the change of color jitter to be small.

We first padded each image by black bands on four sides, this step makes the image larger and thus helps avoid clipping flakes in the following rotation step. This rotation step randomly chooses a rotating degree uniformly from 0 to 360 degrees. After that, flip the image horizontally. Not all images are invariant to horizontal flipping, however, in our application it is a valid tool. In general, horizontal flipping is used much less than vertical. Finally, color, brightness, and hue of the image is changed by sampling corresponding changing factors uniformly from [0.9, 1.1]. For each image, we generated 30 independent augmented images, which enlarges data size by 30 times. When combining with the quantization method, we quantized the augmented images.

Color Quantization

We employed color quantization using k-means from scikit-learn 1.1.1. The code utilized for this process was slightly modified by removing nested for loops and using torch.randperm from torch.tensor instead of sklearn.shuffle when fitting on a small section of the image.

Tree-based Methods Features

Images were recreated with a selected number of k-means color centroids. All possible differences in these color centroids were placed into bins. The color difference between two colors was calculated as the square root of the squared difference between their RGB values. The following outlines this binning process. The lowest bin contained flake color differences that ranged from the lowest one calculated to 5. From there the color differences are binned in increments of 10 until an upper range of 140. A manual sampling of randomized images indicated that a flake to background differences would not exceed 140 therefore we selected a large bin for this upper range (140-180). The final bin encompasses all other color differences (140 – max color difference). The lower ranges effectively distinguished images that contained mainly background and thus have very similar colors creating consistently small differences. Color differences in middle ranges indicated the presence of flakes. Large color differences implied high amounts of bulk material. Specifically all calculated color ranges were: [minimum color difference - 5], [5 - 10], [10 - 20], [20 - 30], [30 - 40], [40 - 50], [50 - 60], [60 - 70], [70 - 80], [80 - 90], [90 - 100], [100 - 110], [110 - 120], [120 - 130], [130 - 140], [140 - 180], [180 - maximum color difference]. To prevent extreme overfitting of the tree-based classifiers when determining accuracy and confusion matrices, we only used the ranges [5,10], [60-70] and [180 – maximum color difference]. When creating receiver operating characteristic curves all ranges were used to better understand the estimators’ sensitivity and specificity.

Figure 1: Detailed outline of the algorithm used to extract features from two-dimensional matrix representation of input images which are used for tree-based methods during classification.

Tree-based Methods Grid Search Cross-Validation

To determine the best hyperparameters for each tree-based method (decision tree, boosted decision tree, random forest) we employ the scikit learn GridSearchCV algorithm. This method performs an exhaustive search for optimization of an estimator’s parameters by cross-validation over a parameter grid. For the decision tree estimator, the parameter grid consisted of the tree’s max depth varying from one to 10 and its max leaf nodes varied from one to 10. For the boosted decision tree, we varied tested learning rates of 0.001, 0.01, and 0.1 and varied the tree’s max depth from one to 10. For the random forest, the max depth varied from four to 10 and the number of n-estimators varied from one to 10.

Masking Algorithm

We created a masking algorithm which when given an image with a thin flake will return an image with the flake in white and everything else in black. The algorithm accepts user input to select the flake in the image. We employed this masking algorithm for a post processing of images that the CNNs correctly classified.

Confusion Matrices and Receiver Operating Characteristic Curves

The confusion matrices indicated that the tree-based algorithm successfully filtered out images without flakes while mainly misclassifying images with flakes as not containing them. Through manual identification, these false negatives usually contained very small pieces of flakes. However, in practice, a dataset of images of a slide containing a fabricated flake will contain a large majority of images without flakes rendering the algorithm’s ability to filter out these images extremely useful.

Furthermore, the receiver operating characteristic (ROC) curves demonstrated that the tree-based algorithms can be tuned to have high true positive rates at the expense of moderately high false positive rates. This way the algorithm can accurately identify images with flakes and an experienced user can quickly go through these images to find the false positives. For example, in the ROC curve with 256 colors the random forest can achieve a true positive rate of about 80% and a false positive rate of about 20%. For example, if there are 100 images a realistic distribution would be 80 images with no flake and 20 with a flake. Once this dataset has been run through the tree-based algorithm, 16 images would be true positives and 16 images would be false positives. One would only have to go through 32 images (about a third of the original dataset) to find 80% of the flakes. In practice, this will rapidly speed up flake identification. Overall, both confusion matrices and ROC curves demonstrated that the ensemble algorithms performed with higher success than the single decision tree.

Figure 2: Confusion matrices for tree-based algorithms. Confusion matrices for tree-based classifiers when images are recreated with 5, 20, and 256 colors.

Figure 3: Receiver operating characteristic (ROC) curves. ROC curves for each tree-based classifier when images are recreated with 5, 20, and 256 colors.

Classifier Accuracy on Subset Training

Deep-learning algorithms require large datasets while tree-based methods do not. To evaluate the effect of training dataset size on all algorithms, we trained the estimators with subsets of the training data after a standard 75/25 train test split. We trained on 10% and 50% of the available training data (75% of the original dataset). The accuracy of the CNNs was determined by their performance on the test data (25% of the original dataset). The tree-based methods were optimized with a grid search and 5-fold cross-validation. Once the hyperparameters were determined, the estimator’s accuracy was determined from training and testing on available data. The CNNs accuracy decreased substantially with less training data while the tree-based methods maintained their performance.


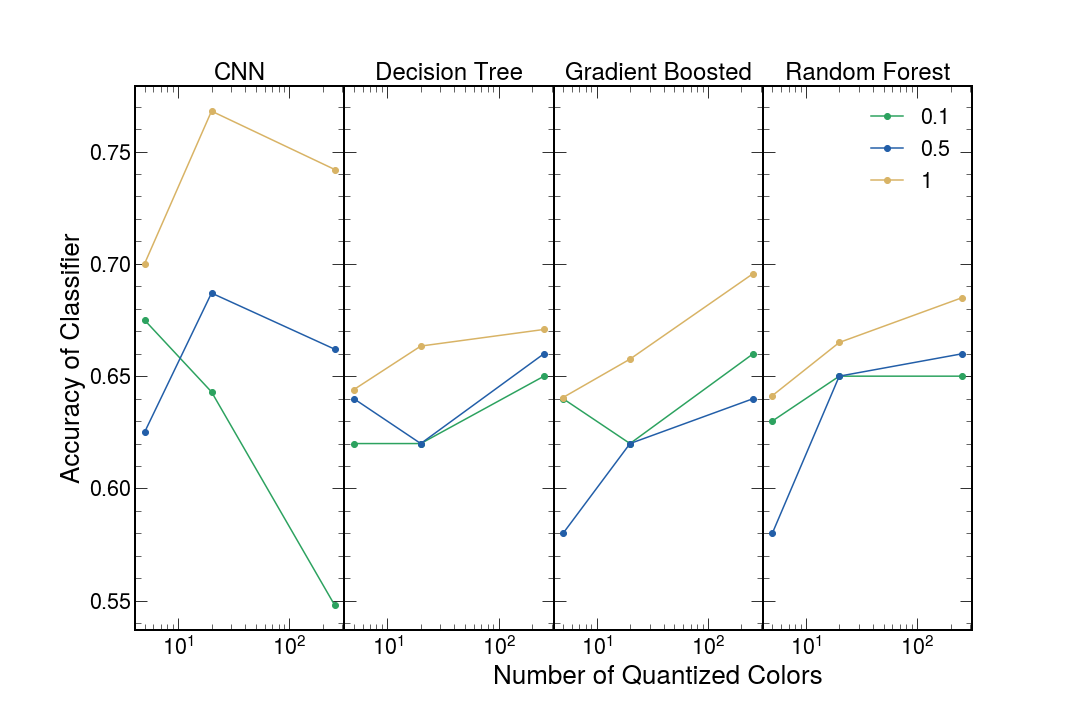


Figure 4: Accuracy of CNNs and tree-based methods when training on data subsets. CNNs’ (first panel) and tree-based methods’ (last three panels) test accuracies when trained with 10%, 50% and 100% of available training data from a 75/25 train test/split on entire data set.

**Table S1. Tree-based estimators’ performance with 10% of training data and varied quantized colors**

|  | **Quantized Colors** | | | | | | | | | |
| --- | --- | --- | --- | --- | --- | --- | --- | --- | --- | --- |
|  | **5** | | | **20** | | | | **256** | | |
|  | **Train** | **Test** | **Hyperparameters** | **Train** | **Test** | **Hyperparameters** | **Train** | | **Test** | **Hyperparameters** |
| Decision Tree | 0.66 | 0.62 | Max depth: 4  Max leaf: 4 | 0.79 | 0.62 | Max depth: 6  Max leaf: 4 | 0.87 | | 0.65 | Max depth: 5  Max leaf: 7 |
| Gradient Boosted Decision Tree | 0.68 | 0.64 | Max depth: 2  Learning rate: 0.001 | 0.79 | 0.62 | Max depth: 3  Learning rate: 0.001 | 0.87 | | 0.66 | Max depth: 3  Learning rate: 0.001 |
| Random Forest | 0.72 | 0.63 | Max depth: 4  N-estimators: 4 | 0.80 | 0.65 | Max depth: 4  N-estimators: 4 | 0.87 | | 0.65 | Max depth: 4  N-estimators: 4 |

Fractional train and test accuracy and associated hyperparameters for tree-based estimators trained with 10% of available training data from a 75/25 train/test split.

**Table S2. Tree-based estimators’ performance with 50% of training data and varied quantized colors**

| **Classifier** | **Quantized Colors** | | | | | | | | | |
| --- | --- | --- | --- | --- | --- | --- | --- | --- | --- | --- |
|  | **Five** | | | **20** | | | | **256** | | |
|  | **Train** | **Test** | **Hyperparameters** | **Train** | **Test** | **Hyperparameters** | **Train** | | **Test** | **Hyperparameters** |
| Decision Tree | 0.61 | 0.64 | Max depth: 5  Max leaf: 4 | 0.72 | 0.62 | Max depth: 5  Max leaf: 4 | 0.74 | | 0.66 | Max depth: 5  Max leaf: 4 |
| Gradient Boosted Decision Tree | 0.65 | 0.58 | Max depth: 7  Learning rate: 0.1 | 0.71 | 0.62 | Max depth: 3  Learning rate: 0.001 | 0.77 | | 0.64 | Max depth: 4  Learning rate: 0.001 |
| Random Forest | 0.65 | 0.58 | Max depth: 10  N-estimators: 10 | 0.73 | 0.65 | Max depth: 5  N-estimators: 7 | 0.78 | | 0.66 | Max depth: 5  N-estimators: 7 |

Fractional train and test accuracy and associated hyperparameters for tree-based estimators trained with 50% of available training data from a 75/25 train/test split.

**Table S3. Tree-based estimators’ performance with all training data and varied quantized colors**

| **Classifier** | **Quantized Colors** | | | | | | | | | |
| --- | --- | --- | --- | --- | --- | --- | --- | --- | --- | --- |
|  | **Five** | | | **20** | | | | **256** | | |
|  | **Train** | **Test** | **Hyperparameters** | **Train** | **Test** | **Hyperparameters** | **Train** | | **Test** | **Hyperparameters** |
| Decision Tree | 0.62 | 0.64 | Max depth: 5  Max leaf: 4 | 0.68 | 0.66 | Max depth: 7  Max leaf: 7 | 0.73 | | 0.67 | Max depth: 7  Max leaf: 7 |
| Gradient Boosted Decision Tree | 0.61 | 0.64 | Max depth: 4  Learning rate: 0.001 | 0.67 | 0.66 | Max depth: 4  Learning rate: 0.001 | 0.74 | | 0.70 | Max depth: 7  Learning rate: 0.001 |
| Random Forest | 0.62 | 0.64 | Max depth: 5  N-estimators: 7 | 0.68 | 0.67 | Max depth: 5  N-estimators: 7 | 0.73 | | 0.69 | Max depth: 5  N-estimators: 7 |

Fractional train and test accuracy and associated hyperparameters for tree-based estimators trained with 100% of available training data from a 75/25 train/test split.

**Table S4. CNNs’ performance with varied training data and quantized colors**

| **Training Data Used During Training** | | | | | | |
| --- | --- | --- | --- | --- | --- | --- |
|  | **10%** |  | **50%** |  | **100%** |  |
| **Quantized Colors** | **Train** | **Test** | **Train** | **Test** | **Train** | **Test** |
| 5 | 1.0 | 0.68 | 1.0 | 0.63 | 1.0 | 0.70 |
| 20 | 1.0 | 0.64 | 1.0 | 0.69 | 1.0 | 0.77 |
| Infinite (all) | 0.96 | 0.54 | 1.0 | 0.67 | 1.0 | 0.74 |

Fractional train and test accuracy for CNNs’ from 75/25 train/test split.

Light Intensity Distribution of Raw Images

The images used for the training and testing datasets used throughout this manuscript were collected at various microscope settings. These settings change the amount of light exposure or perceived brightness of each image. This created a more diverse dataset of images used for training and testing. Below we showcase images with the lowest, moderate, and highest amount of light exposure. The values for the brightness of each image represent the arithmetic mean pixel level for the first band in the image.

Figure 5: Histogram of the distribution of brightness of raw images used for training and testing all algorithms. A selection of images with high, moderate, and low brightness are also displayed with their corresponding brightness values.

Train and Test Time of CNNs and Tree-Based Methods

Here we examine the amount of time required to fit and evaluate the CNNs and tree-based models. The time to evaluate the model includes the time it takes the model to classify training and testing data and determine the accuracy rate. As can be seen by the tables below, the tree-based methods represent significantly quicker and thus more accessible methods. All codes were run on Google Colab and the specifications of this environment are shown below (S7).

**Table S5. Time required to fit and evaluate the tree-based estimators**

| **Classifier** | **Five Quantized Colors** | | | |
| --- | --- | --- | --- | --- |
|  | **Fit Time (sec)** | **Train Time (sec)** | **Test Time (sec)** | |
| Decision Tree | 0.060 | 0.0050 | | 0.0031 |
| Gradient Boosted Decision Tree | 2.8 | 0.048 | | 0.0071 |
| Random Forest | 0.075 | 0.010 | | 0.0051 |

Time required to fit and determine training and testing accuracies for each tree-based classifier.

**Table S6. Time required to fit and evaluate the CNNs**

| **CNN** | **Five Quantized Colors** |
| --- | --- |
| Fit Time  (hr:min:sec) | 04:00:56 |
| Train Time  (hr:min:sec) | 00:31:08 |
| Test Time  (hr:min:sec) | 00:13:54 |

Time required to fit and determine training and testing accuracies for the CNNs.

**Table S7. Computer specifications**

| **Google Colab** | |
| --- | --- |
| CPU | Intel(R) Xeon(R) CPU @ 2.20GHz |
| CPU Cache size | 39424 KB |
| No. CPU Cores | 2 |
| GPU | Nvidia K80 |
| GPU Memory | 12 GB |
| Performance | 4.1 TFLOPS |
| Available RAM | 12.5 GB |

Computer specifications for Google Colab which all codes were run on for time comparisons.
